# Chitosan Biocomposites for the Adsorption and Release of H_2_S

**DOI:** 10.3390/ma14216701

**Published:** 2021-11-07

**Authors:** Mary Batista, Moisés L. Pinto, Fernando Antunes, João Pires, Silvia Carvalho

**Affiliations:** 1 Centro de Química Estrutural, Faculdade de Ciências da Universidade de Lisboa, 1749-016 Lisboa, Portugal; mkbatista@fc.ul.pt (M.B.); fantunes@fc.ul.pt (F.A.); 2 CERENA, Departamento de Engenharia Química, Instituto Superior Técnico, Universidade de Lisboa, 1049-001 Lisboa, Portugal; moises.pinto@tecnico.ulisboa.pt

**Keywords:** hydrogen sulphide (H_2_S), zeolites, activated carbon, glycerin, chitosan, adsorption

## Abstract

The search for H_2_S donors has been increasing due to the multiple therapeutic effects of the gas. However, the use of nanoporous materials has not been investigated despite their potential. Zeolites and activated carbons are known as good gas adsorbents and their modification with chitosan may increase the material biocompatibility and simultaneously its release time in aqueous solution, thus making them good H_2_S donors. Herein, we modified with chitosan a series of A zeolites (3A, 4A and 5A) with different pore sizes and an activated carbon obtained from glycerin. The amount of H_2_S adsorbed was evaluated by a volumetric method and their release capacity in aqueous solution was measured. These studies aimed to verify which of the materials had appropriate H_2_S adsorption/release properties to be considered a potential H_2_S donor. Additionally, cytotoxicity assays using HeLa cells were performed. Considering the obtained results, the chitosan composite with the A zeolite with the larger pore opening was the most promising material to be used as a H_2_S donor so a further cytotoxicity assay using H_2_S loaded was conducted and no toxicity was observed.

## 1. Introduction

Since its discovery by Carl Wilhelm Scheele in 1775 [1] research on hydrogen sulphide has been changing. Initially, it was mainly focused on its toxicology [2,3,4] and methods for separating it from gas mixtures [5]. The detection of endogenously produced H_2_S in the brain tissues of mammals in 1989 and the paper of Abe and Kimura suggesting that endogenous H_2_S plays a functional role in the regulation of neuronal function [6,7,8] redirected the research to the potential physiological and pathophysiological role of H_2_S. Currently, its therapeutic effects are recognized and exogenous H_2_S exerts cytoprotective and anticancer effects, promotes wound healing, inhibits platelet aggregation and protects against myocardial ischemia, among others [9,10]. However, the main challenge remains the effective exogenously delivery of H_2_S. The direct use of gas or sulphide salts has many drawbacks such as poor dose control leading to toxicity and difficulty in storing and handling gas at high pressures. Developing small molecules that trigger H_2_S after a specific stimulus (hydrolyses, thiol, light and enzymes) was an attempt to achieve controlled H_2_S release in biological conditions. However, their application has found some drawbacks, such as the fast diffusion of the molecules after administration that causes systemic delivery or the formation of by-products that may be toxic or responsible for the therapeutic effect, and poor water solubility [11,12]. Although some of those small molecules showed potential, some obstacles to their practical application have been observed. For instance, garlic and garlic-derived sulphur compounds are known to have protective effects in biological systems; however, it is claimed that this effect is from sulphane sulphur compounds and not from hydrogen sulphide [13,14] and the same was observed with p-hydroxybenzothioamide (thiol activated H_2_S donor). The amount of H_2_S released by this donor is so little that more investigations are needed to clarify if the observed biological activities may be attributed to H_2_S [13,15]. Another work involving Lawesson’s reagent demonstrated that it reduced the severity of colitis, yet in addition to the poor solubility of the Lawesson’s reagent the H_2_S release mechanism involved the hydrolysis reaction of the reagent, leading to an uncontrollable release of H_2_S [16]. Additionally, the synthesis of those donors may have several reaction steps, which may impede its use. Comprehensive reviews concerning H_2_S donors may be found [11,13,17], in which the great prevalence of homogeneous donors is evidenced. The use of porous materials as H_2_S donors has been less explored despite it being a promising strategy that may help to overcome some of the problems observed with homogeneous donors (synthesis, unknown reaction mechanism and formation of by-products). The high surface area of porous materials led to a high payload of the gas, yet its release may be faster than desired [12]. Surface modification of the material can be a strategy to overcome this limitation [18]. Among the high diversity of polymers that may be used for this purpose, chitosan—a biodegradable polymer (polysaccharide) with many biological applications (inhibition of tumour cells, antifungal properties, acceleration of wound healing) [19]—has been used in the modification of materials for several purposes [20,21]. Although its potential research concerning H_2_S adsorption and its release in the liquid phase by porous materials is very scarce [22].

Here, the H_2_S donors’ capacities of several materials, zeolites (3A, 4A, 5A), crystalline hydrated aluminosilicate, with different pore openings of 3, 4 and 5 Å and an activated carbon (obtained from glycerin) and their chitosan composites, were evaluated. The selection of the materials allowed us to observe the influence the *A*_BET_ surface areas, the cation in the structure’s and material’s chemical nature have on their H_2_S adsorption/release capacity as well as on the synthesis of chitosan biocomposites.

The demonstration of the biocompatibility was assessed with cytotoxicity assays using HeLa cells for all the chitosan composites. The biocomposite 5A@Chi, the material that showed the best H_2_S adsorption/release proprieties, was loaded with H_2_S and cytotoxicity assays were also performed. 

## 2. Materials and Methods

All chemicals were commercial and used as received. Zeolites (3A, 4A and 5A) from BDH—Laboratory Reagent, sulfuric acid 96%, acetic acid glacial, low molecular weight chitosan, sodium sulphide nonahydrate ≥ 99.99%, and Teflon (poly(tetrafluoroethylene)) particle size 35 μm from Sigma-Aldrich, a mixture of industrial crude glycerin (82% glycerol, from a Portuguese company) and 5,5′-Dithiobis(2-nitrobenzoic Acid) > 98% from TCI.

The HeLa (human cervical cancer cell line) cells for the cytotoxicity studies were from the American Type Culture Collection, Manassas, VA, USA. The medium RPMI-1640 without L-glutamine from Corning Inc. Penicillin-streptomycin, L-glutamine, foetal bovine serum (FBS), trypsin (2.5%, without phenol red), were from Thermo Fisher Scientific (Manassas, VA, USA).

### 2.1. Materials

The glycerin-based activated carbon (Gta@600) was prepared by a combination of acid carbonization and thermal activation, as described in detail elsewhere [23]. Briefly, the glycerin@char (G@char) was prepared by hydrothermal synthesis using a mixture of glycerin and sulfuric acid in a volume ratio of 1:0.5. The mixture was transferred to a Teflon-lined stainless-steel autoclave and the acid carbonization was made at 180 °C for 6 h in an oven (Medline Scientific Limited, model ON-02G) pre-heated to the desired temperature. The G@char was washed with distilled water until reaching pH 7 and was dried. The thermal activation of G@char under a N_2_ flow of 5 cm/min at 600 °C for 1 h led to the activated carbon (Gta@600).

Chitosan-based materials were synthesized using a methodology adapted from the literature [24]. Briefly, the chitosan (0.5 g) was dissolved in 1 wt. % of acetic acid solution (50 mL). Then, after complete dissolution (24 h), this mixture was added to a suspension containing 2 g of materials (zeolites or activated carbon) and 40 mL of water and it was stirred for 24 h. The mixture solution obtained was washed three times by centrifugation at 1400× *g* with distilled water, and was dried overnight at 50 °C.

### 2.2. Materials Characterization

Fourier-Transform Infrared (FTIR) spectra were acquired in KBr pellets using a Nicolet 6700 FTIR spectrometer between 4000 and 400 cm^−1^ (64 scanning; 4 cm^−1^ resolution). The samples were sputtered-coated with a gold/palladium alloy (80/20 wt.%) (5–10 nm thick) and the morphology of the powers was analysed by Scanning Electron Microscopy (SEM) performed on a Zeiss Supra 55 VP apparatus using 5 kV as the accelerating voltage. An X-ray diffractometer (Pan Analytical PW3050/60X’Pert PRO) was used to acquire the XRD patterns in the range of 5–60° (2θ) with CuKα radiation (λ = 0.15406 Å). Elemental analysis was carried out in a CHNS Analyzer (Thermofinnigan Flash, EA, 1112 series). Oxygen content for the activated carbons was obtained by the difference between the total percentage (100%) and the sum of percentage (wt.%) of carbon, hydrogen, sulphur, and nitrogen.

Nitrogen gas adsorption–desorption isotherms were measured at −196 °C in a constant volume adsorption automated apparatus (Quantacrome, Nova 2200e). Prior to measurements, about 50 mg of each sample was degassed under a vacuum of 0.133 Pa at 150 °C for 2 h and 120 °C overnight for zeolites and activated carbons materials, respectively. The N_2_ isotherms data were used to estimate the apparent area, *A*_BET_, and to evaluate it through the BET equation (0.05 < p/p0 < 0.15) and ISO 92777 [25,26]. The microporosity was analysed by NLDFT (non-local density functional theory) model, using the N_2_—silica equilibrium transition kernel at 77K based on a cylindrical pore model provided by NovaWin version 10.0 software. Thermogravimetry coupled with Differential Scanning Calorimetry (TG-DSC) data were obtained using equipment from Setaram (mod. TG-DSC 111). Experiments were carried out under air flux with a temperature ramp of 5 °C/min from ambient to 600 °C.

### 2.3. Hydrogen Sulphide Adsorption Studies

Gas-solid hydrogen sulphide adsorption isotherms (Air Liquide) were obtained at low relative pressures, with ≈ 60–100 mg of sample, by volumetric method. The sample temperature (25 °C) was maintained with a water bath (Sub Aqua 2 Plus, Grant). Sample outgassing was carried out in a vacuum greater than 10^−2^ Pa, for 2.5 h at 150 °C. This method is described in detail in References [27,28,29] and a schematic representation of the apparatus and of the methodology used are shown in the Supporting Materials (Figure S1.1).

### 2.4. Hydrogen Sulphide Release in Aqueous Solution

The H_2_S release from the materials was followed in aqueous solution by UV-Vis using the DTNB (5,5′-Dithiobis (2-Nitrobenzoic Acid)) based on a methodology previously reported [30]. The reaction involved is shown in Equation (1). Briefly, 1L of a DTNB solution (0.116 mM) was prepared by dissolving 46 mg of DTNB (>98% TCI) in 1L of PBS (phosphate buffer solution) at pH 7.2 prepared in mili-Q water. Before the release studies, a calibration curve was performed using the DTNB solution and a Na_2_S·9H_2_O solution freshly prepared. Aliquots of 10 μL of the Na_2_S solution (2.1 mM) were added to a cuvette having 3 mL of DTNB solution; 2 min after each addition the UV-Vis spectrum was taken between 250–550 nm. The obtained spectra and calibration curve are presented in the Supplementary Materials (Figure S2.1).



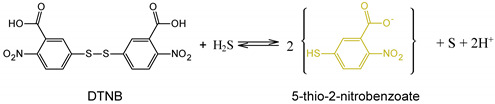

(1)


The release studies were conducted by adding the H_2_S loaded samples to the DTNB solution followed by stirring. The kinetic curves were obtained with a UV-Vis spectrophotometer (Genesys 10S UV-Vis Spectrophotometer from Thermo Scientific Blank) at room temperature. The first spectrum was acquired after 2 min of sample addition, followed by 15 min intervals until no changes were observed in the spectra. To prevent sample dispersion in the liquid phase, the materials were mixed with Teflon particles, in a wt.% ratio of 75:25 (sample:Teflon) to form pellets as described in [22,31]. The quantity of DTNB solution used was dependent on the material adsorption and release capacity and was such that it was in excess to assure that all the H_2_S released was quantified and produced considerable changes in the UV-Vis spectrum without saturating it. These conditions were achieved by using 2.5 mL of DTNB solution for Gta@600 (m_pellets_ = 6.2 mg), Gta@600Chi (m_pellets_ = 6.1 mg), 3A@Chi (m_pellets_ = 8 mg) and 4A@Chi (m_pellets_ = 5.4 mg) and 50, 150, 100 and 60 mL for 3A (m_pellets_ = 4.1 mg), 4A (m_pellets_ = 2mg), 5A (m_pellets_ = 4.1 mg) and 5A@Chi (m_pellets_ = 3 mg), respectively. The pellet was H_2_S loaded by introducing it into a small glass basket inside a glass container with a PTFE vacuum valve. The container was connected to the vacuum line, and samples were outgassed, as stated before. After the material returned to room temperature, H_2_S was introduced and was left equilibrating overnight. After evacuation, to remove the excess of H_2_S, the container was filled with helium until atmospheric pressure.

### 2.5. In vitro Cytotoxicity Studies

Cytotoxicity studies were performed using HeLa cells due to their broad use as a first assessment of materials’ toxicity. Before the assays, the cell culture was incubated at 37 °C in a humidified atmosphere with 5% CO_2_ and was left to grow until ≈ 70–80% subconfluency in the RPMI-1640 medium with 10% (*v*/*v*) foetal bovine serum (FBS), antibiotics (100 UI/mL penicillin and 100 μg/mL streptomycin) and 2 mM glutamine. To this end, cells were seeded in 96-well plates (2500 cells/well for the 72 h experiment and 7500 cells/well for the 24 h experiment) or in 12-well plates (112.5 cell/well for 48 experiment) for the loaded 5A@Chi studies. After 24 h incubation, the medium was replaced with fresh media containing the materials in suspension at 450 μg/mL for the materials’ cytotoxicity assays or solid H_2_S loaded 5A@Chi (1 mg/well or 4.5 mg/well) for release studies

Cell viabilities were determined by adding 10 μL/well or 200 μL/well of Alamar-Blue solution in 96 well plates and 12 well plates, respectively, and were incubated for 4 h at 37 °C. A change in the solution colour from blue to pink allows the quantification of the live cells by fluorescence (λ_ex_ = 560 nm, λ_em_ = 590 nm). The relative cell viability (%) was calculated using untreated cells as a control and was calculated as follows (Equation (2)):

cell viability (%) = [fluorescence average]_sample_/[fluorescence average]_control_ × 100 (2)

Data are expressed as mean value ± standard deviation (n = 3). Statistical significance was calculated using analysis of variance ANOVA.

## 3. Results

### 3.1. Characterization of the Materials

**Figure 1:**
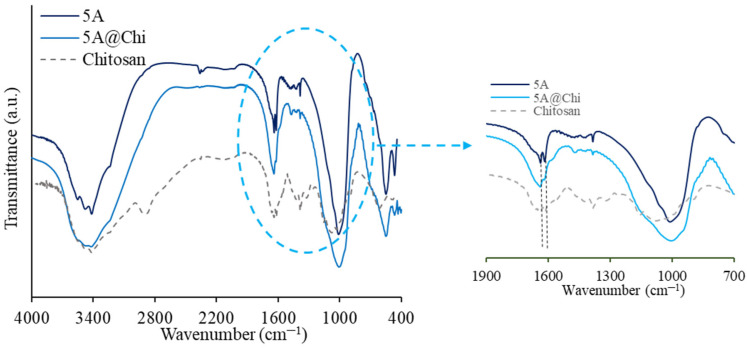
FTIR spectra of the indicated samples. An ampliation of the spectrum is shown in the right side.

Different techniques were used for the characterization of the chitosan-modified materials such as Fourier-Transform Infrared (FTIR) spectroscopy, X-ray powder diffraction (XRD), Scanning Electron Microscopy (SEM), thermogravimetric analysis (TG-DSC), and elemental analysis. The results suggested that, although the chitosan was present in all the composites, the synthetic procedure led to some unexpected results in some of the studied zeolites. In fact, for 5A@Chi, all the data indicated the presence of the chitosan without the damage of the material’s surface. The FTIR spectra of the 5A@Chi (Figure 1) showed a broader band at ≈3600–3300 cm^−1^ in comparison with the 5A zeolite, which may be attributed to hydrogen-bonded O–H stretching overlapped with the several N–H stretching bands present in the chitosan. The bands in ≈1600 cm^−1^ zone may be attributed to the chitosan by its amide characteristic bands, with the C=O amide I and N–H bending amide II appearing at ≈1640 cm^−1^, and ≈1610 cm^−1^, respectively [19], but also to the stretching and bending vibration of the hydroxyl groups in the zeolites (1630 cm^−1^) [19,32]. The bands at ≈1415 cm^−1^ (C–N stretching coupled with N–H plane deformation) and ≈1390 cm^−1^ C–N stretching of the amino groups at 1325 cm^−1^ of chitosan were observed. Finally, the Si–O–Si and the Al–O–Si bending vibrations were observed at ≈440 cm^−1^ and 550 cm^−1^, respectively.

CHNS elemental analyses were also performed, and the results are shown in Table 1. The amount of C present in the 5A@Chi comes exclusively from the chitosan and confirms its presence yet is in low quantities. The fact that the N in the zeolites’ biocomposites could not be determined may be explained by its proportion in the chitosan. The elemental analysis of chitosan showed that the C proportion is 5.3 times more than N, so since chitosan is present in low quantities in the zeolites its determination is not possible [33].

**Figure 2:**
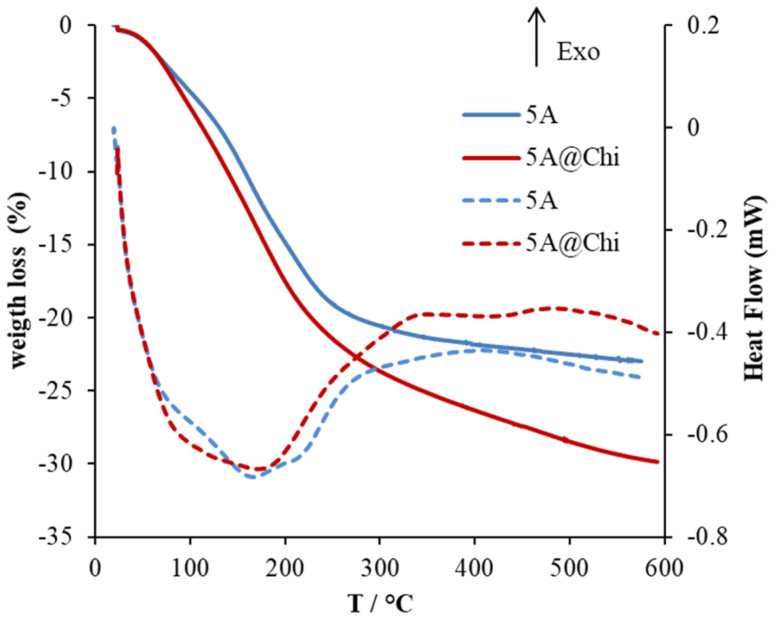
TG (solid lines) and DSC (dashed lines) data for the indicated samples.

The TG-DSC curves for 5A zeolite with and without chitosan (Figure 2) revealed initially (below 225 °C) a strong contribution for the mass decreases due to the water loss, corresponding to an endothermic peak in the DSC curve. After about 225 °C, the DSC signal is endothermic and can be ascribed to the polymer decomposition, that is, the oxidation of the organic molecules of chitosan in agreement with the data documented in the literature [34,35,36], indicating that the decomposition of chitosan, in various atmospheres, occurs mainly between 225 and 525 °C. In this way, Table 2 shows the amount of chitosan estimated by subtracting, for a respective material, the mass losses in the temperature range of 225–525 °C of the material with and without chitosan.

**Figure 3:**
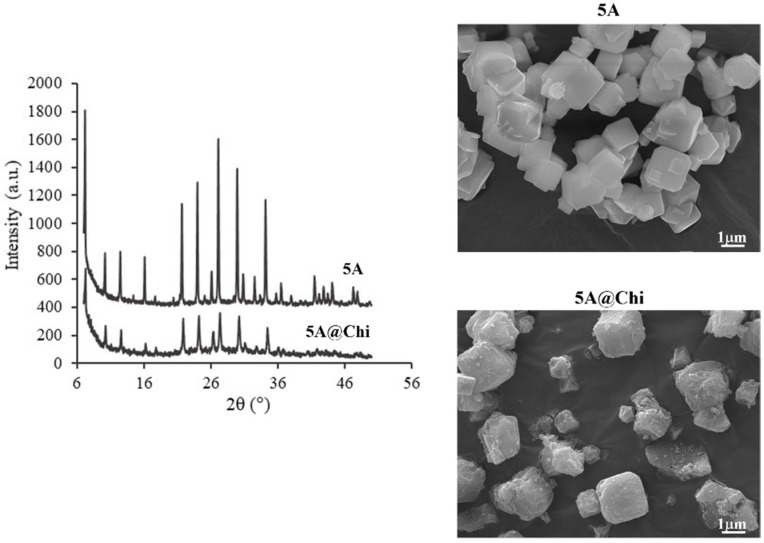
Powder XRD patterns (**left**) and SEM images (**right**) of the 5A zeolite and 5A@Chi.

Figure 3 shows the XRD and SEM images of the 5A zeolite and 5A@Chi. The XDR pattern of 5A@Chi shows only a reduction in the reflection peak’s intensity in comparison with 5A, which may result from hydrogen bonds between chitosan and zeolite [37]. The SEM images show the representative well-defined cubic shape with a homogeneous and smooth surface of the type A zeolites [35] concurrently with the presence of chitosan in their surfaces.

**Figure 4:**
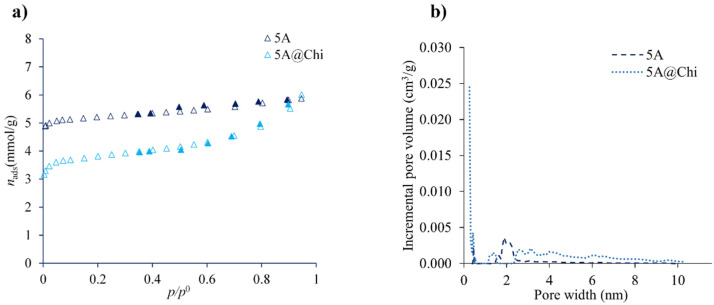
Nitrogen adsorption-desorption isotherms (**a**) and corresponding pore size distribution curves (**b**) of 5A zeolite and 5A@Chi materials.

The N_2_ adsorption-desorption isotherms at −196 °C of zeolites (5A and 5A@Chi) are shown in Figure 4a. The original zeolite (5A) exhibited a Type I isotherm with a small H4 hysteresis loop, as often found for zeolites [28]. For the biocomposite (5A@Chi), the isotherm is of the same type as for the parent material, but the H4 hysteresis loop [28] is now more evident, as a more probable consequence of the chitosan deposition. The latter observation agrees with some (although low) degree of mesoporosity that is formed upon the modification of the zeolite with chitosan, as can be noticed from the pore-size distribution in Figure 4b. The obtained *A*_BET_ surface areas, in the context of the present work, are regarded as an apparent surface as indicated by IUPAC [28], for 5A and 5A@Chi were of 409 m^2^ g^−1^ and 301 m^2^ g^−1^, respectively, which corresponds to a decrease of ≈26%, suggesting that chitosan is partially covering the pores. If the reduction in the microporous volumes (obtained by the t-method [28]) is considered, from 0.167 to 0.102 cm^3^ g^−1^ for 5A and 5A@Chi, respectively, the decrease is even higher (39%).

For the other zeolites composites (3A@Chi and 4A@Chi), the TG-DSC (Figure S3.1), SEM images (Figures S4.1 and S4.2) and elemental analysis gave similar results to the 5A@Chi mostly due to the nature of the techniques, it being possible to assert by the elemental analysis (% carbon) (Table 1) and TG-DSC (Table 2) that the presence of chitosan was more expressive in the 4A@Chi composite and less for the 3A@Chi. Nevertheless, the FTIR spectra and the XDR pattern revealed unexpected results. The FTIR spectra (Figures S5.1 and S5.2) showed the same bands as 5A@Chi, an exception was made for the Al–O–Si bending vibrations, which was not present while, in the XDR (Figures S4.1 and S4.2), the reflection peaks were broadened suggesting the loss of the crystalline domain. These observations led to the fact that there would be any aspect in the synthetic procedure that could explain this evidence, then we decided to treat the 4A zeolite only with a solution of acetic acid 1 wt.% without chitosan (4A@Ac). The FTIR spectrum had substantial changes when compared with the untreated 4A zeolite, one of them being the disappearance of the Al–O–Si bending vibrations (Figure S5.3), suggesting that the acetic acid solution affected the crystal structure of 4A zeolite. The XDR pattern also shows broadened reflection peaks (Figure S4.3). In fact, this phenomenon has already been mentioned by Kyotani et al. [38], who noticed acetic acid may damage the surface of NaA zeolite by way of the dissolution of sodium and silicon affecting the crystal structure and elemental composition.

The 4A@Chi N_2_ isotherms (Figure S6.1) presented a Type II isotherm with a surface area of 48 m^2^ g^−1^; the 4A@Ac showed a Type I isotherm with an *A*_BET_ of 110 m^2^ g^−1^ (Figure S6.2), while the 4A zeolite had *A*_BET_ of 363 m^2^ g^−1^ [37]. This high decrease in the area of 4A@Chi (≈87%) and 4A@Ac (≈70%) in comparison with the 4A zeolite confirms that the acetic acid solution affects the 4A zeolite structure and the presence of chitosan.

Finally, the Gta@600 and Gta@600Chi FTIR spectra (Figure S5.4) display the glycerin-carbons bands of symmetric (971–1220 cm^−1^) and asymmetric (1384 cm^−1^) stretching modes of −SO_3_H groups. The amides bands of the glycerin-carbons (1637 cm^−1^ and 1614 cm^−1^) could also be observed, which overlap the amides regions of the chitosan. No relevant information could be retrieved (Figure S4.4) for Gta@600 and Gta@600Chi from XDR since both materials showed broad reflections, indicating the amorphous patterns, as expected for this type of carbon material [39].

The TG-DSC analysis (Figure S3.1) indicated that a similar situation occurs initially for the carbon material (Gta@600) in comparison with the zeolite´s composites, although the total mass loss at the end is more pronounced due to the decomposition of the carbon matrix itself. The elemental analysis (Table 1) of the Gta@600 and Gta@600Chi shows a decrease in the carbon quantity due to the presence of chitosan. This may be related to the increase in the oxygen content that is present in the chitosan polymer.

Finally, the Gta@600 displayed a Type I isotherm corresponding to microporous materials, which was further confirmed by the DFT pore-size distribution in (Figure S6.3a,b)). The Gta@600Chi exhibited an isotherm characteristic of a non-porous material and with low surface area (<5 m^2^ g^−1^), as a most probable consequence of the extensive coverage of the porosity by the chitosan. In fact, as shown in Table 2, the Gta@600Chi was the material with the highest chitosan amount.

### 3.2. Hydrogen Sulphide Adsorption Isotherms

The H_2_S adsorption data show that, among the composites, only the 5A@Chi presented some adsorption capacity. For the others it became negligible. These results corroborate the N_2_ isotherms data, namely, those obtained for Gta@600Chi, indicating the formation of a non-porous material, suggesting that the introduction of the polymer in the Gta@600 blocked the access to the pores; whereas, for 4A@Chi, besides this pore blocking event it may also result from the surface damage caused by acid acetic solution as discussed before.

**Figure 5:**
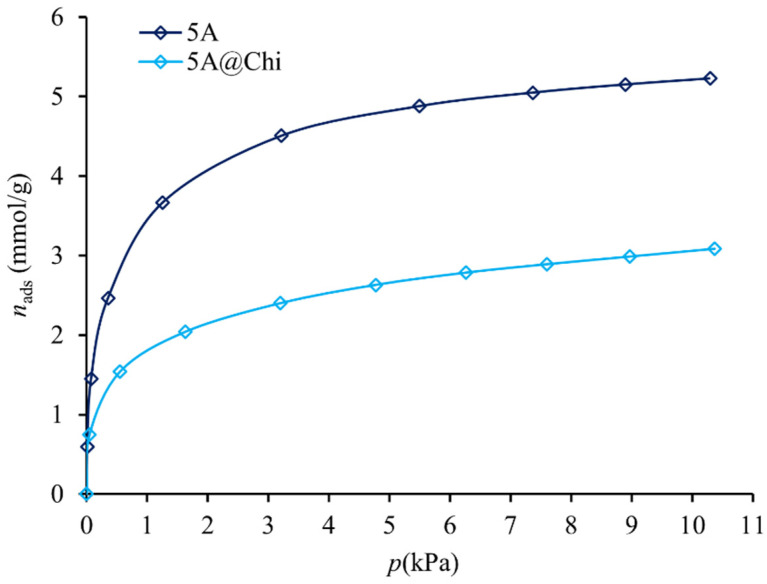
H_2_S adsorption isotherms of the 5A zeolite and 5A@Chi.

Analysing the parent materials, in the case of 4A and 5A zeolites (Figure S7.1), with pore openings of 0.38 and 5 nm [40], respectively, hydrogen sulphide is expected to enter the structure (Figure S7.2) as seen by the amounts adsorbed in Figure 5 and Figure S1.2. Following the same reasoning, the 3A zeolite has a pore opening of 0.3 nm [40], thus H_2_S molecules (kinetics diameter is 0.36 nm [41]) do not easily access the porosity of the material, making the adsorption kinetics too slow. This explains why no isotherm was obtained for the latter.

Among all the parent materials, 5A zeolite has the higher adsorption capacity for hydrogen sulphide, followed by 4A zeolite and activated carbon. Although the *A*_BET_ of the activated carbon (466 m^2^ g^−1^) is higher than for 5A zeolite (409 m^2^ g^−1^), it has a lower adsorption capacity, suggesting that, besides the apparent surface area, the interactions with the surface, namely between the cations in the zeolite and the H_2_S dipole (0.98 D [42]), can also play an important role. On the other hand, the higher adsorption of 5A in comparison with 4A may be attributed, besides the higher *A*_BET_, to the presence of the Ca^2+^ cation in its structure, which is known to increase the H_2_S adsorption capacity on LTA zeolites [43].

Additionally, the adsorption capacity of chitosan for the H_2_S was evaluated (Supplementary Materials Figure S1.3) and no significant adsorption was observed.

### 3.3. Hydrogen Sulphide Release in Aqueous Solution

**Figure 6:**
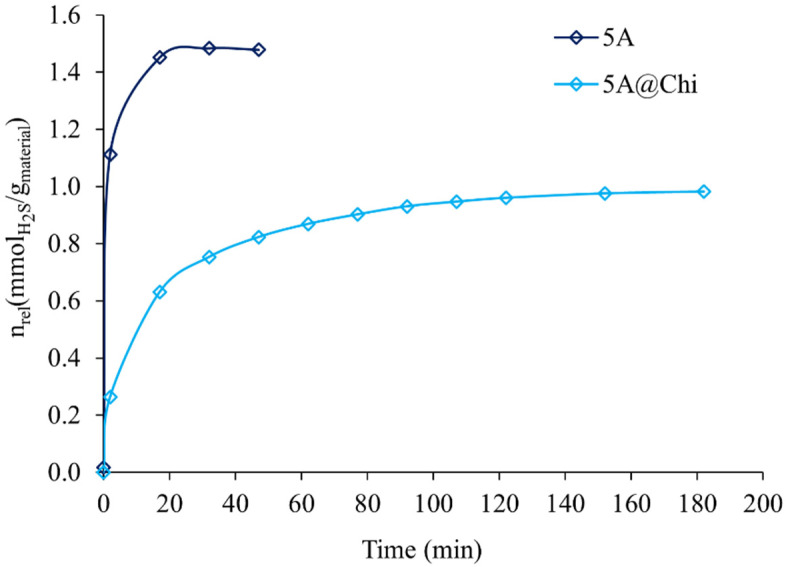
H_2_S release curves in aqueous solution using the DTNB method for the 5A and 5A@Chi.

The H_2_S release in aqueous solution at pH=7.2 was very fast for all the studied materials (≈17 min) except for 5A@Chi (Figure 6 and Supplementary Materials Figure S2.2). In this material, after an initial burst (17 min) corresponding to the release of ≈64% of the total gas released, a still release until 120 min was observed. Additionally, the amount of H_2_S released by the composites was also negligible (except for 5A@Chi) and is in line with the marked reduction in the porosity, as already discussed. Additionally, analysing Table 3 it is also possible to observe that the materials released only a small part of the adsorbed gas. In the zeolites, this may be explained by the dissociation of H_2_S, by its coordination to the cations and/or by hydrogen-bond interactions with the framework oxygen or with SiOHAl [41]. The fact that 5A zeolite has Ca^2+^ ion in its framework leading to a stronger interaction with H_2_S, allied with its larger micropore size and volume allowing a deeper position inside the pores, may explain the amount released by 5A zeolite, since in the literature the dissociative H_2_S adsorption is mostly reported to occur with Na^+^ cations [43]. Finally, for 5A@Chi, a slow release was observed, indicating chitosan was able to delay the release of the gas, having reached the balance between the adsorbed and release kinetics. Although this is an important result, it is still behind the existing homogenous materials, which have longer releasing times, yet some of them, such as a phosphonamidothioate-based molecules, have a similar peak time (10 min) [44,45].

Regarding the activated carbon, the H_2_S released (Figure S2.2) was insignificant, which may be explained by material–H_2_S interaction. In fact, it is recognized that these interactions are very complex in activated carbons [46] and a more extended degradation of H_2_S may happen. Furthermore, in the release curve of Gta@600 after a plateau in absorbance had been reached, a decrease was observed due to the adsorption by the Gta@600 of the 5-thio-2-nitrobenzoate anion formed during the reaction (Equation (1)).

### 3.4. Cytotoxicity Results

**Figure 7:**
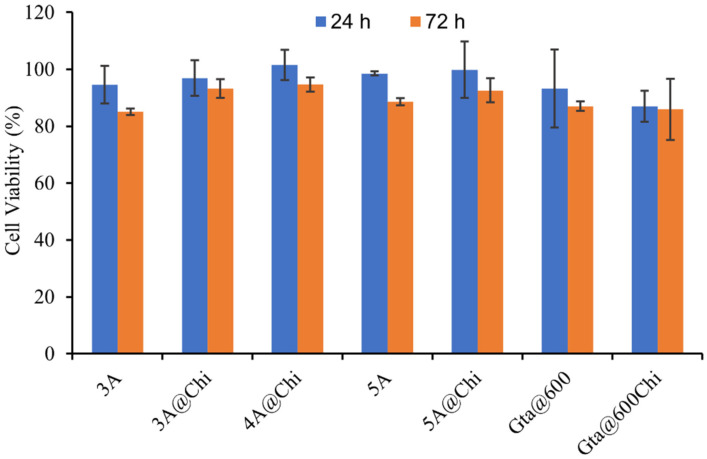
HeLa viability in the presence of unloaded materials. Materials toxicity was assessed after 24 h (blue bars) and 72 h (orange bars) in contact with cells, using a material concentration of 450 μg/mL. Each bar represents an average (of 3 independent experiments each one with 8 replicates) ± SD.

HeLa cells were used for the cytotoxicity assay and a high material concentration of 450 μg/mL was chosen (Figure 7), since no toxicity is expected from those materials [41]. Zeolites 3A and 5A showed no toxicity, as observed previously for 4A zeolite [17]. The biocomposites showed no toxicity to the cells, as expected, since chitosan is a biocompatible polymer (Figure 7). The observed small decrease in the cell viability of Gta@600Chi when compared with Gta@600 had no significance and was within the experimental error.

**Figure 8:**
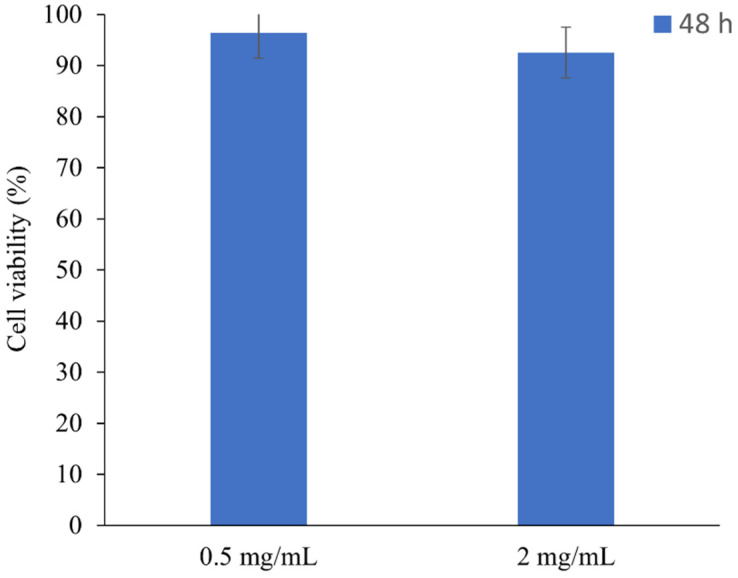
HeLa viability in the presence of loaded materials. Materials toxicity was assessed after 48 h in contact with cells, using a material concentration of 0.5 mg/mL and 2.0 mg/mL. Each bar represents an average (of 3 independent experiments each one with 2 replicates) ± SD.

To understand whether the loaded material remains not toxic to the cells, 0.5 mg/mL and 2 mg/mL of the most promising material (5A@Chi) was loaded with H_2_S and added to the HeLa cells. Both concentrations, which correspond to a H_2_S release of 0.49 μM and 2.20 μM, respectively, showed no toxicity after 48 h Figure 8.

## 4. Conclusions

The characterization results showed that, although the synthetic procedure was the same for all the materials, the obtained results are highly dependent on the zeolite type, and a damage of the 4A zeolite surface due to the acetic acid having been observed.

The 5A zeolite showed a higher adsorption capacity for hydrogen sulphide among all the studied materials due to its higher *A*_BET_ and the presence of Ca^2+^ cations. Although with higher *A*_BET_, the activated carbon had the lower adsorption capacity, which may be explained by the weaker interaction of the gas with the surface. A decrease in the adsorption of biocomposites was observed when compared with the parent materials due to the presence of the chitosan on the surface. The damage in the 4A@Chi zeolites’ surface explained the severe decrease observed in the 4A@Chi adsorption capacity.

The hydrogen sulphide release studies in aqueous solutions showed that the composite 5A@Chi had the longest release rate, approaching 120 min. These results indicate that 5A@Chi has the best balance between hydrogen sulphide adsorption capacity and the release profile. Cytotoxicity assays showed that none of the materials tested were toxic to HeLa cells at the studied concentrations. Additionally, H_2_S loaded 5A@Chi did not show toxicity to cells at the concentrations of 0.5 mg/mL and 2 mg/mL.

## Figures and Tables

**Table 1:** Elemental analysis of the composites.

Sample	N (%)	C (%)	H (%)	S (%)	O (%)
3A@Chi	n.d.	1.0	2.2	n.d.	n.d
4A@Chi	n.d.	1.9	2.4	n.d.	n.d
5A@Chi	n.d.	1.4	2.3	n.d.	n.d
Gta@600Chi	1.0	75	2.1	7	14.9
Gta@600	0.9	80	n.d.	7	12.1

**Table 2:** Mass loss (%) in the range 225–525 °C.

Sample	Parent Material		Material with Chitosan		Chitosan (%)
	225 °C	525 °C	225 °C	525 °C	
3A	16.5	20.7	16.9	26.1	5.4
4A	17	21.6	17.9	28.4	10.5
5A	17.3	22.7	19.8	28.9	9.1
Gta@600	9.1	53.1	8.3	75.6	23.3

**Table 3:** H_2_S released at room temperature using DNB method.

Sample	H_2_S_released_ (mmol_H2S_/g_sample_)	H_2_S_released_ (%)	*t*_max_ (min)
3A	1.60	-	17
3A@Chi	0.02	-	17
4A	1.35	43.8	17
4A@Chi	0.03	18.8	17
5A	1.48	28.3	17
5A@Chi	0.98	31.8	120
Gta@600	0.02	0.94	17
Gta@600Chi	0.012	2.28	17

## Data Availability

Data sharing is not applicable to this article.

## Supplementary Materials

The following are available online at https://www.mdpi.com/article/10.3390/ma14216701/s1, Figure S1.1. Schematic representation of the volumetric apparatus used in this work.; Figure S1.2. H_2_S adsorption isotherms for the indicated samples.; Figure S1.3. H_2_S adsorption isotherm for the indicated samples.; Figure S2.1. UV-Vis spectra and calibration curve obtained using a Na_2_S solution.; Figure S2.2. H_2_S release curves in aqueous solution using the DTNB method for the indicated samples.; Figure S3.1. TG (solid lines) and DSC (dashed lines) data for the indicated samples.; Figure S4.1. Powder XRD patterns (**left**) and SEM images (**right**) of the 3A zeolite and 3A@Chi.; Figure S4.2. Powder XRD patterns (**left**) and SEM images (**right**) of the 4A zeolite and 4A@Chi.; Figure S4.3. Powder XRD pattern of the 4A zeolite treated with a 1 wt% acid acetic solution.; Figure S4.4. Powder XRD patterns of the Gta@600 and Gta@600Chi.; Figure S5.1. FTIR spectra of the indicated samples. An ampliation of the spectra is shown in the right.; Figure S5.2. FTIR spectra of the indicated samples. An ampliation of the spectra is shown in the right.; Figure S5.3. FTIR spectrum of the 4A zeolite treated with a 1wt% acid acetic solution 4A@Ac.; Figure S5.4. FTIR spectra of the indicated samples. An ampliation of the spectra is shown in the right side.; Figure S6.1. Nitrogen adsorption-desorption isotherms (**a**) and corresponding pore size distribution curves (**b**) for mentioned materials.; Figure S6.2. Nitrogen adsorption-desorption isotherms (**a**) and corresponding pore size distribution curves (**b**) for mentioned materials.; Figure S6.3. Nitrogen adsorption-desorption isotherms (**a**) and corresponding pore size distribution curves (**b**) of activated carbon materials.; Figure S7.1. Structure of type A zeolites.; Figure S7.2. Schematic representation of the structure relationship between the zeolite, H_2_S and chitosan.
