# Supplementary material for: Chitosan Biocomposites for the Adsorption and Release of H2S
Source: Materials (Basel). 2021 Nov 7;14(21):6701. doi: 10.3390/ma14216701 (PMC8587643; doi:10.3390/ma14216701)
Supplement: Supplementary file 1 [file materials-14-06701-s001.zip › materials-1448778-supplementary.pdf]

materials

MDPI

Supplementary Materials

Chitosan Biocomposites for the Adsorption and Release of H_2S

Mary Batista [1], Moisés L. Pinto [2], Fernando Antunes [1], João Pires [1*] and Silvia Carvalho [1,2*]

1 Centro de Química Estrutural, Faculdade de Ciências da Universidade de Lisboa, 1749-016, Lisboa, Portugal; mkbatista@fc.ul.pt (M.B.); fantunes@fc.ul.pt (F.A.)
2 CERENA, Departamento de Engenharia Química, Instituto Superior Técnico, Universidade de Lisboa, 1049-001, Lisboa, Portugal; moises.pinto@tecnico.ulisboa.pt
* Correspondence: silvia.carvalho@tecnico.ulisboa.pt (S.C.); Tel.:+351 21750000 jpsilva@fc.ul.pt (J.P.) Tel.:+351 217500903

S1. Hydrogen sulphide isotherms determination

The hydrogen sulphide adsorption isotherms were determined using the volumetric apparatus schematically represented in Figure S1.1

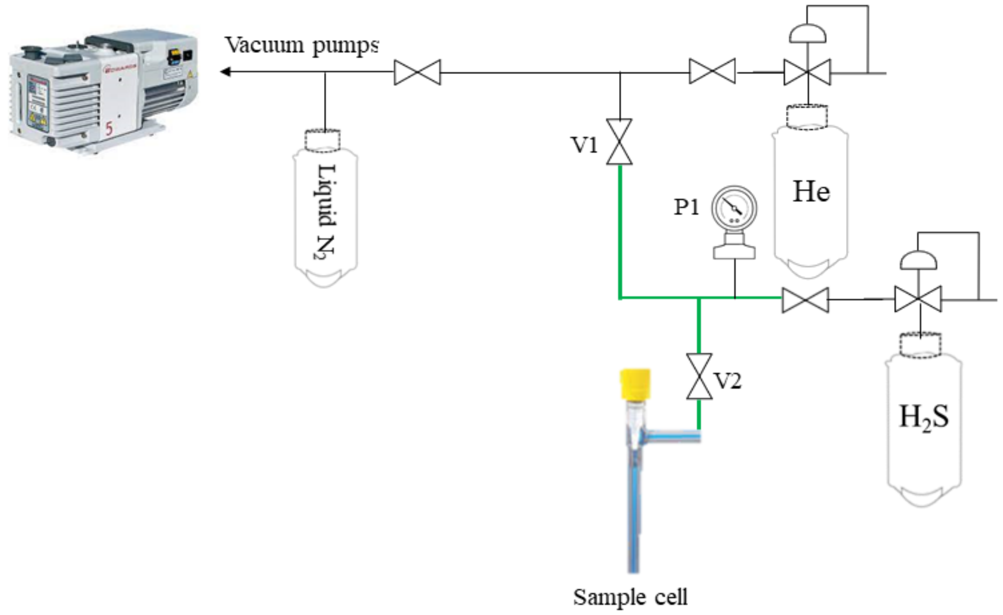

Figure S1.1. Schematic representation of the volumetric apparatus used in this work.

Briefly, a stipulated quantity of H_2S was introduced (measured by the pressure gauge (P1)) in the green zone with the V1 and V2 closed. After the pressure stabilization the V2 was open, and the gas contacts the material in the sample cell till the equilibrium was attained (no change in pressure). The adsorbed amount was calculated using the observed change in pressure and from the application of a gas law. In the present work the gas non-ideality was accounted for by the virial equation of state with the second virial coefficient obtained by interpolation of the values reported in the literature and was under 0.1 % [1].

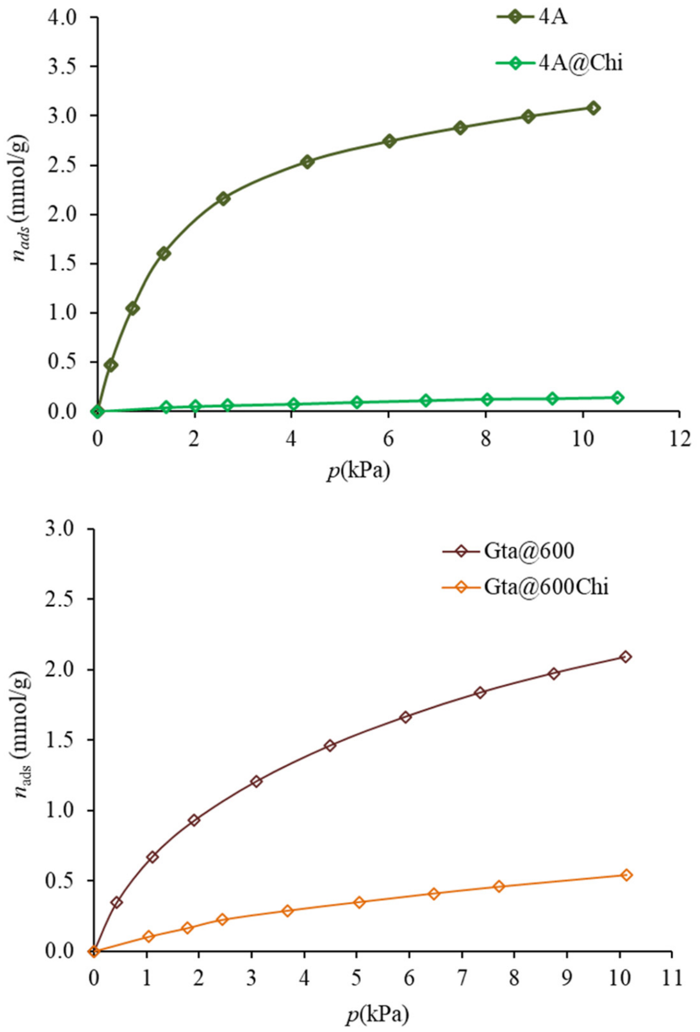

Figure S1.2. H_2S adsorption isotherms for the indicated samples.

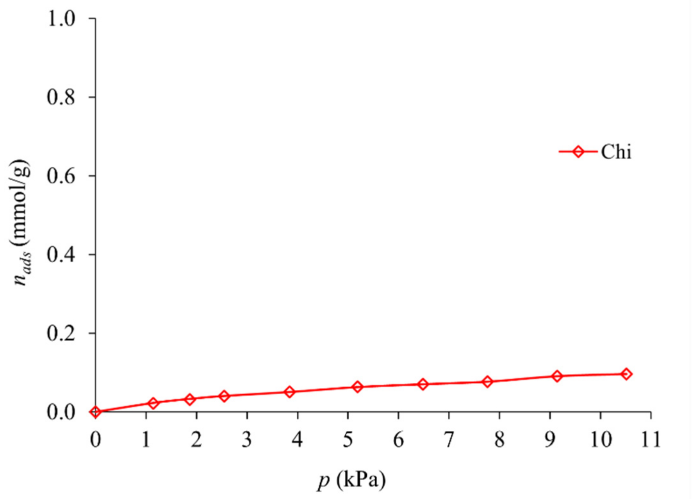

Figure S1.3. H_2S adsorption isotherm for the indicated samples.

S2. Hydrogen sulphide release in aqueous solution

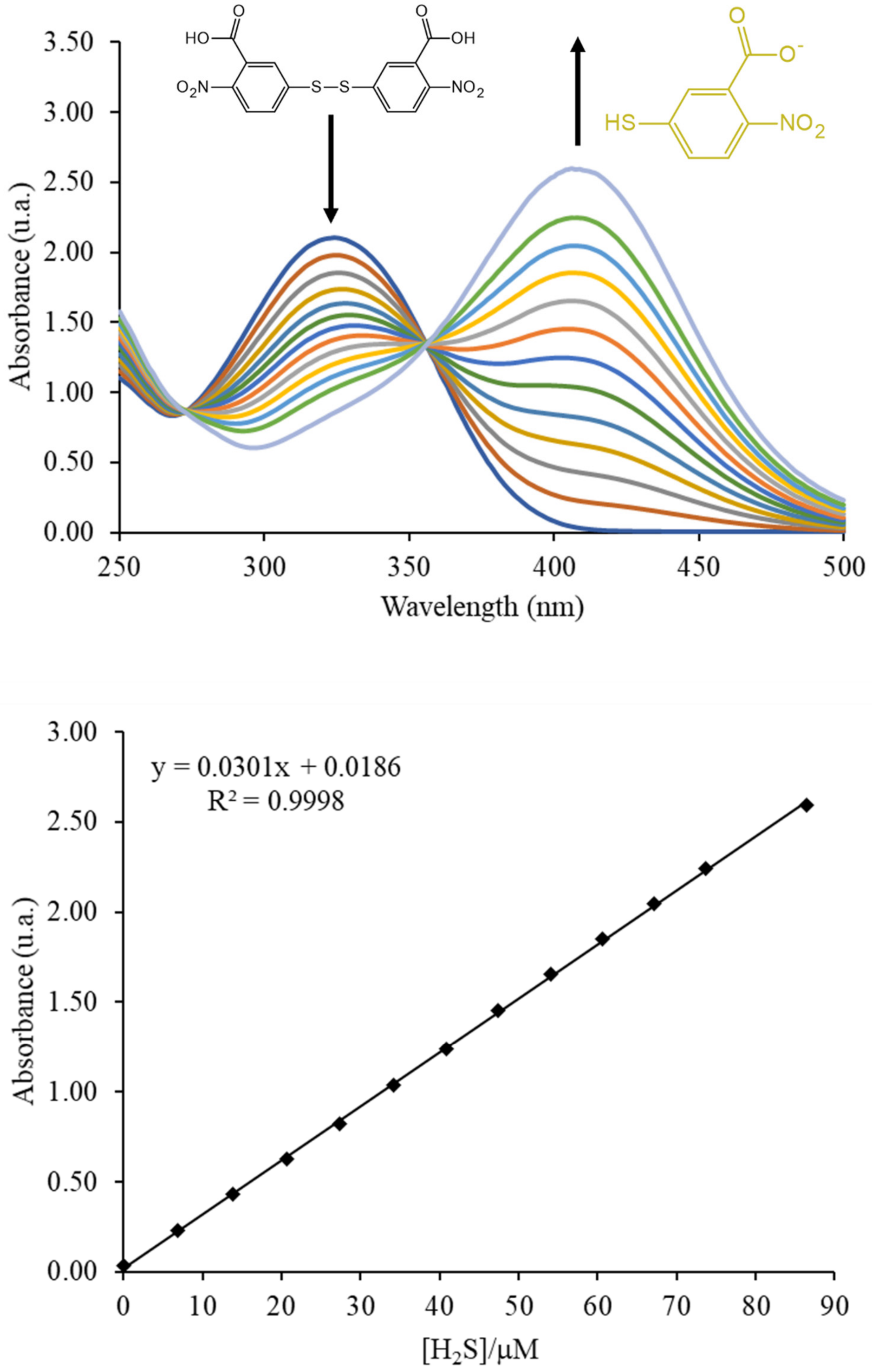

Figure S2.1. UV-Vis spectra and calibration curve obtained using a Na_2S solution.

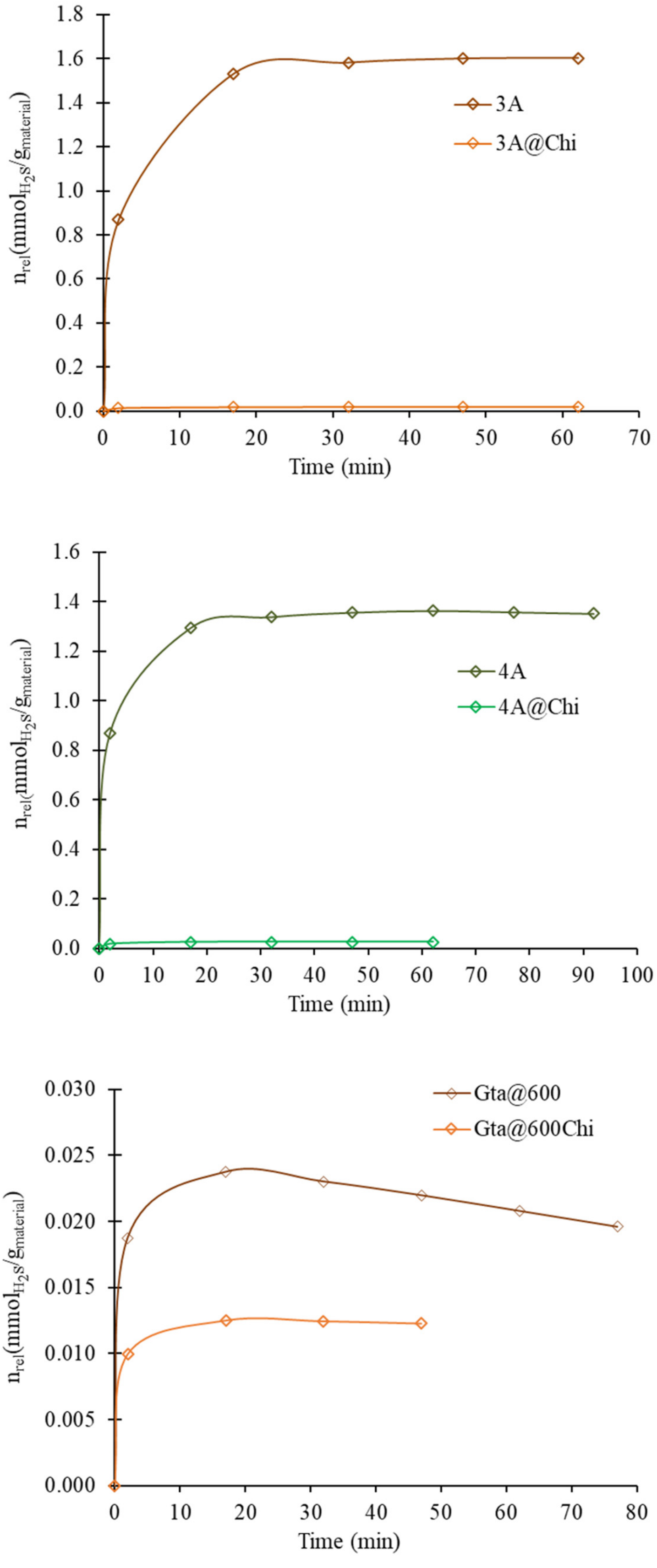

Figure S2.2. H_2S release curves in aqueous solution using the DTNB method for the indicated samples.

S3. TG-DSC data for zeolites 3A and 4A, and activated carbon with and without chitosan

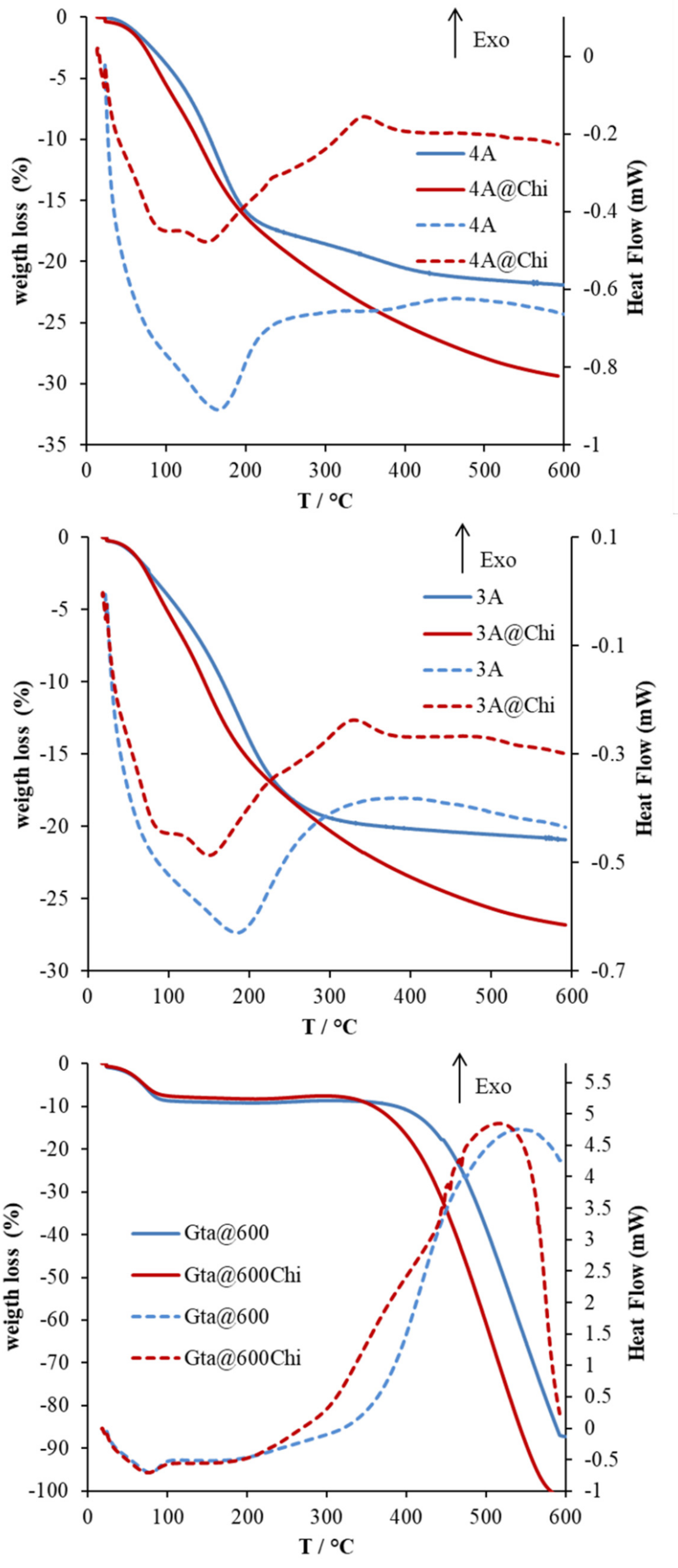

Figure S3.1. TG (solid lines) and DSC (dashed lines) data for the indicated samples.

S4. XRD and SEM data for zeolites 3A and 4A, and activated carbon with and without chitosan

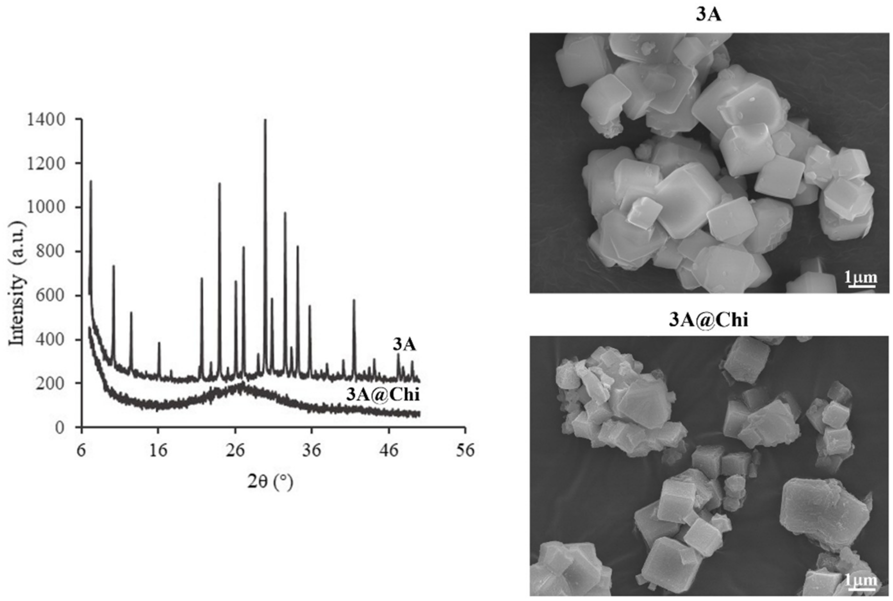

Figure S4.1. Powder XRD patterns (**left**) and SEM images (**right**) of the 3A zeolite and 3A@Chi.

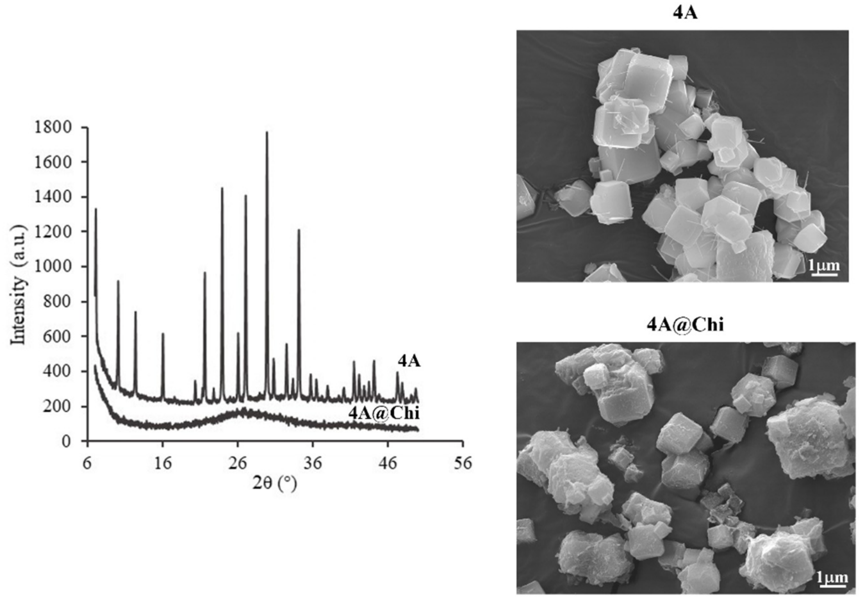

Figure S4.2. Powder XRD patterns (**left**) and SEM images (**right**) of the 4A zeolite and 4A@Chi.

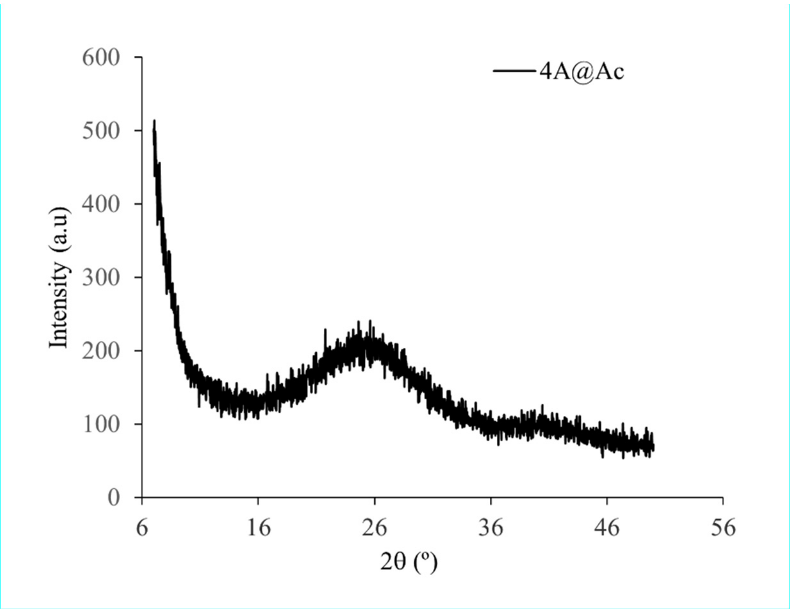

Figure S4.3. Powder XRD pattern of the 4A zeolite treated with a 1 wt.% acid acetic solution.

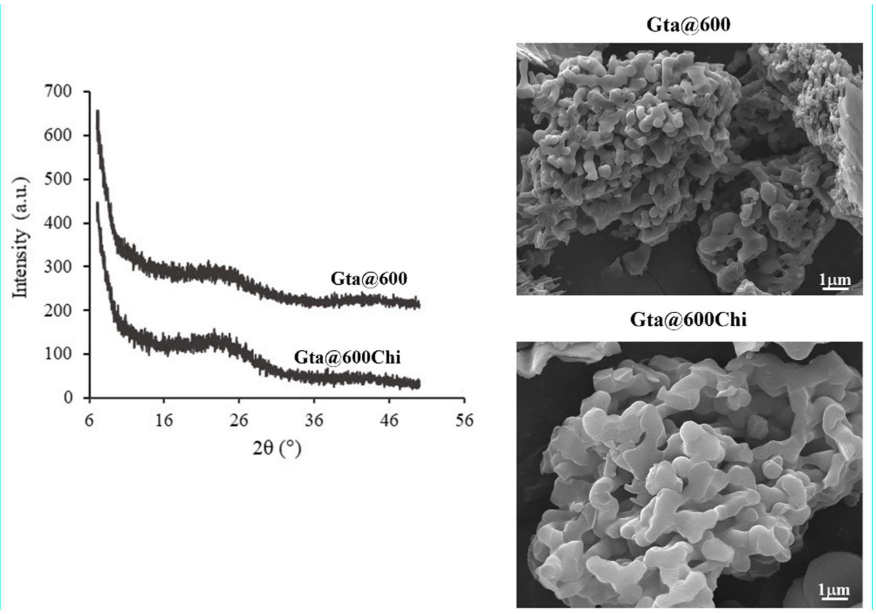

Figure S4.4 Powder XRD patterns (**left**) and SEM images (**right**) of the Gta@600 and Gta@600Chi.

S5. FTIR data for 3A and 4A zeolites and activated carbon, with and without chitosan

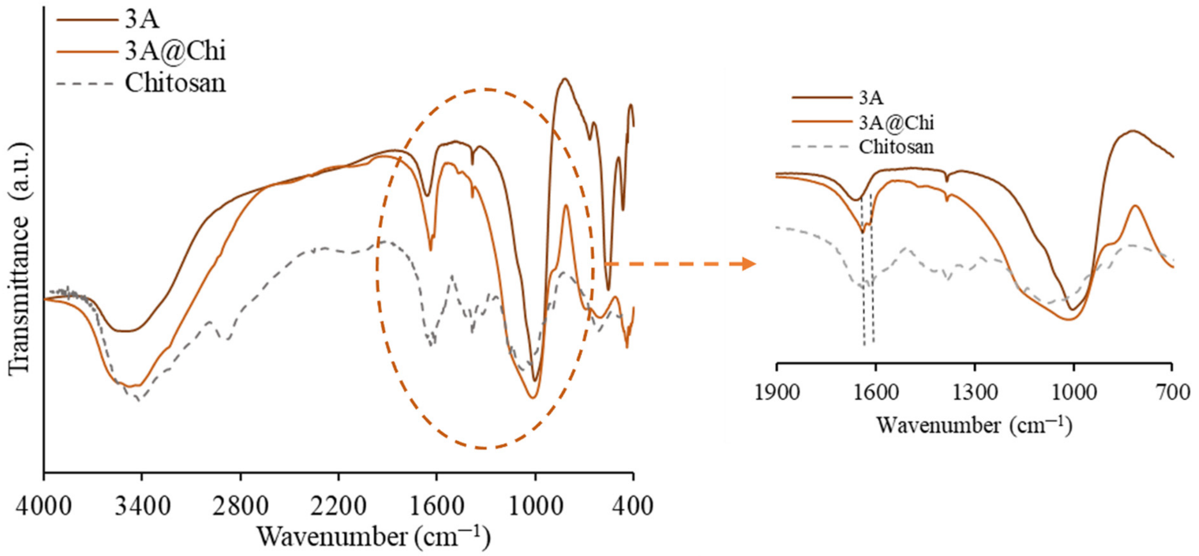

Figure S5.1. FTIR spectra of the indicated samples. An ampliation of the spectra is shown in the right.

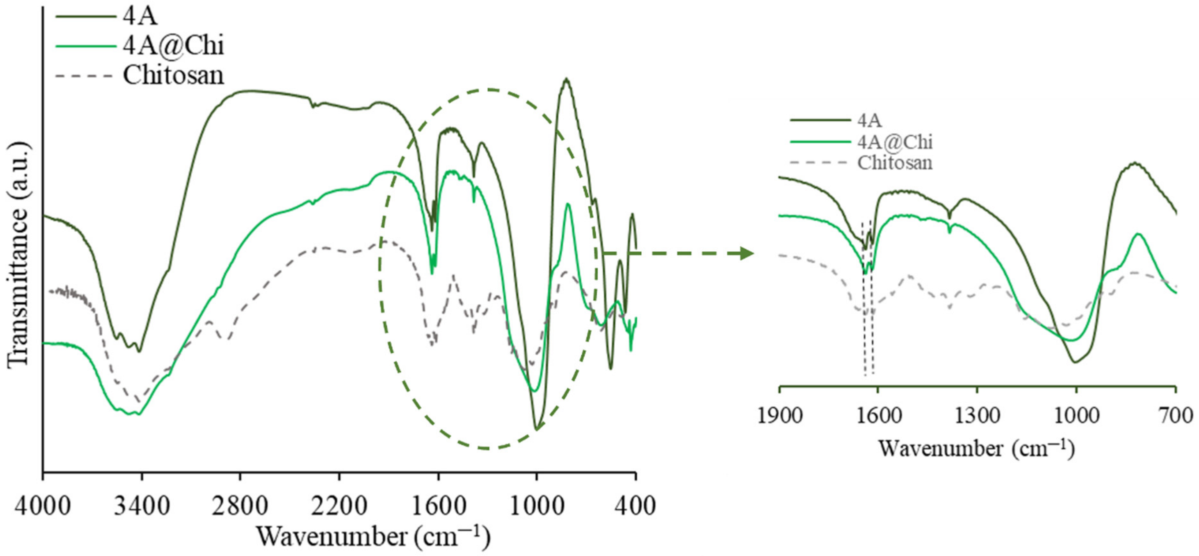

Figure S5.2. FTIR spectra of the indicated samples. An ampliation of the spectra is shown in the right.

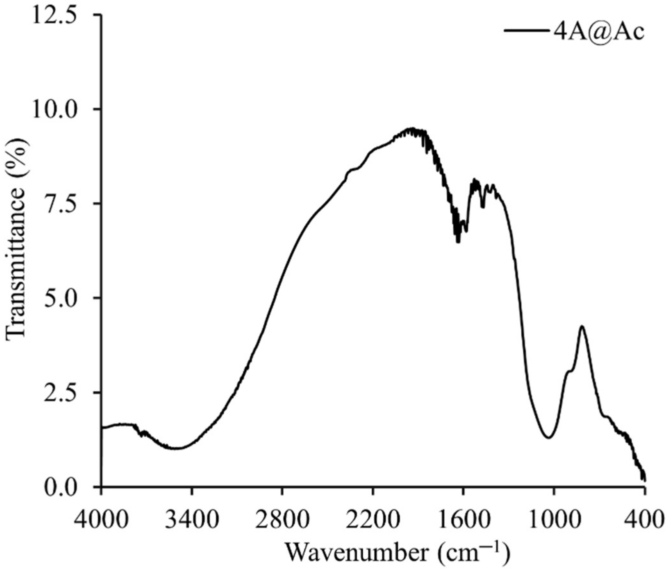

Figure S5.3. FTIR spectrum of the 4A zeolite treated with a 1wt. % acid acetic solution 4A@Ac.

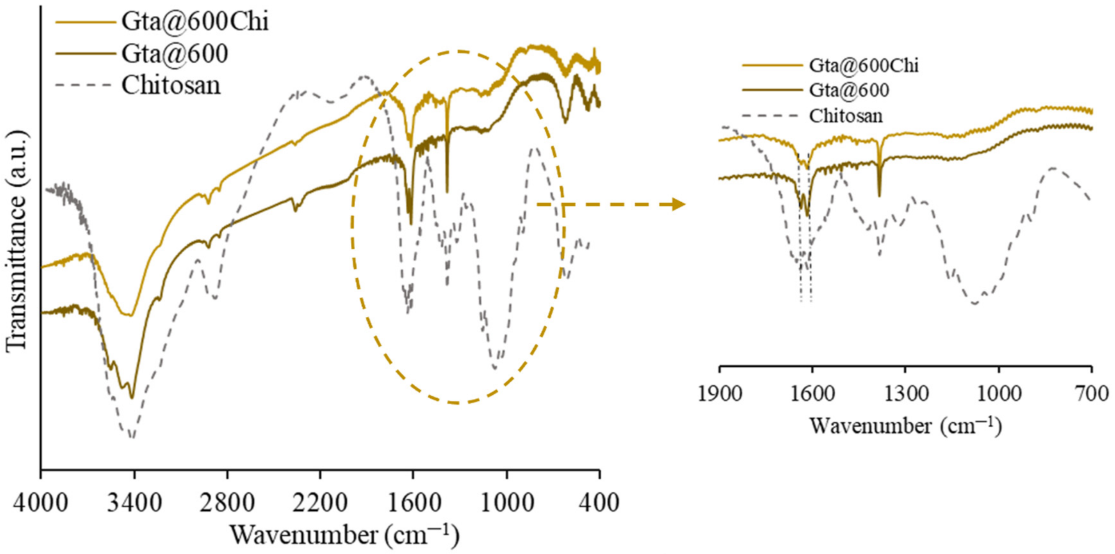

Figure S5.4 FTIR spectra of the indicated samples. An ampliation of the spectra is shown in the right side.

S6. Nitrogen isotherms determination

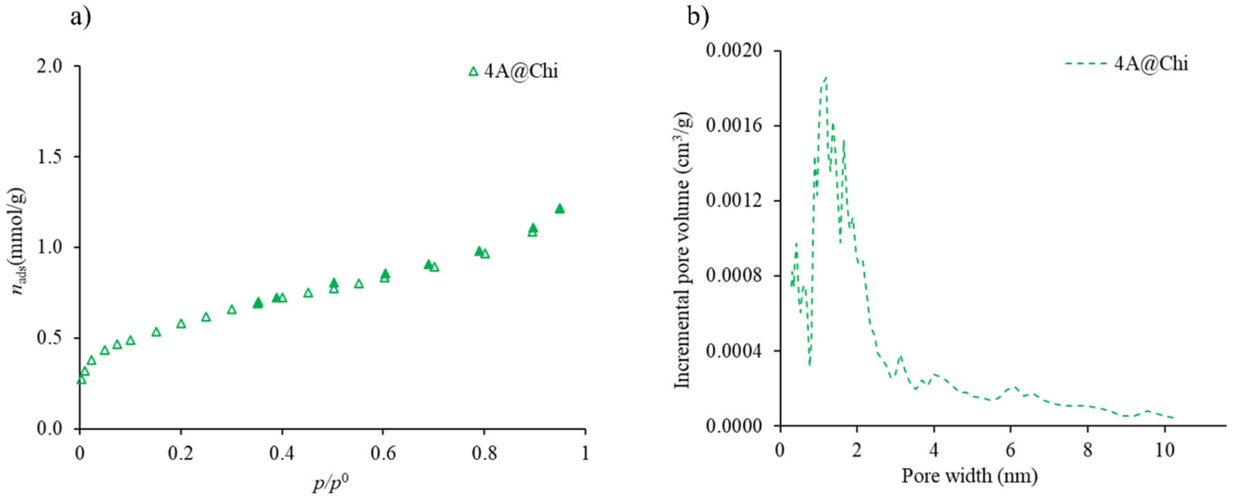

Figure S6.1. Nitrogen adsorption-desorption isotherms (**a**) and corresponding pore size distribution curves (**b**) for mentioned materials.

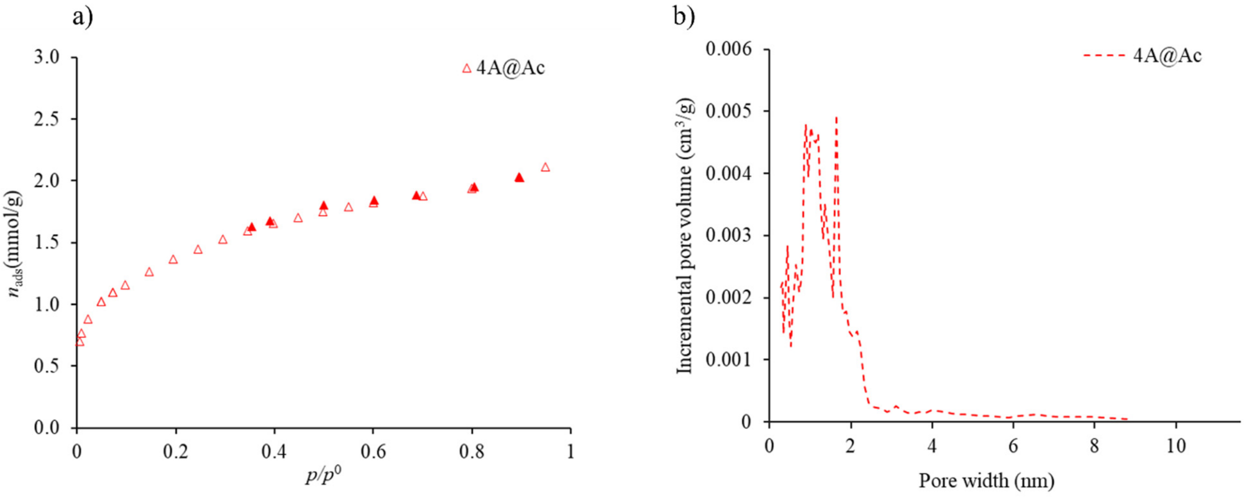

Figure S6.2. Nitrogen adsorption-desorption isotherms (**a**) and corresponding pore size distribution curves (**b**) for mentioned materials.

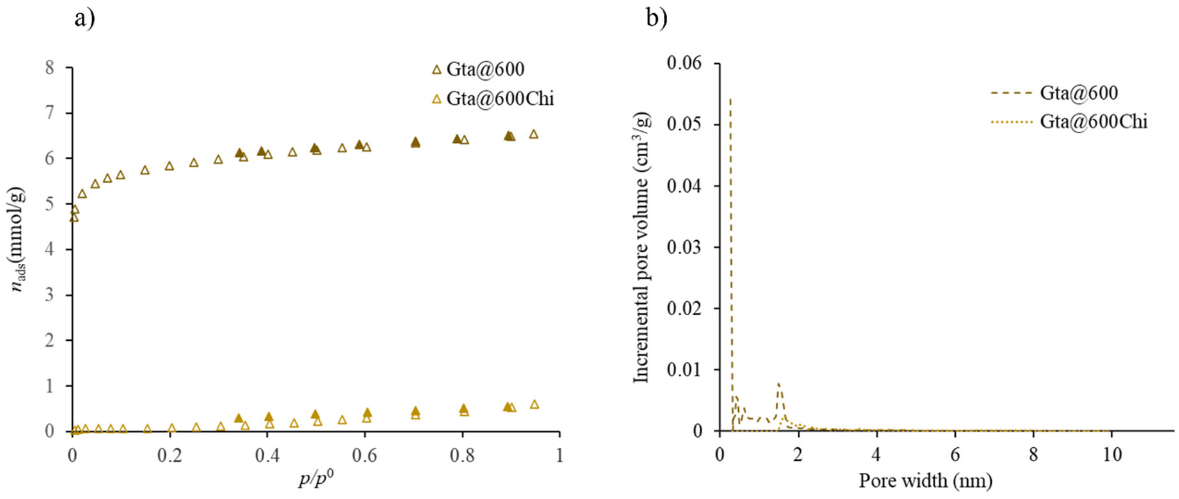

Figure S6.3. Nitrogen adsorption-desorption isotherms (**a**) and corresponding pore size distribution curves (**b**) of activated carbon materials.

S7. Type A zeolites and their structure.

Zeolites are crystalline aluminosilicates porous structures. They are negatively charged, and exchangeable cations are present and balance the charge. Type A zeolite has a cubic structure (Figure S71.1)

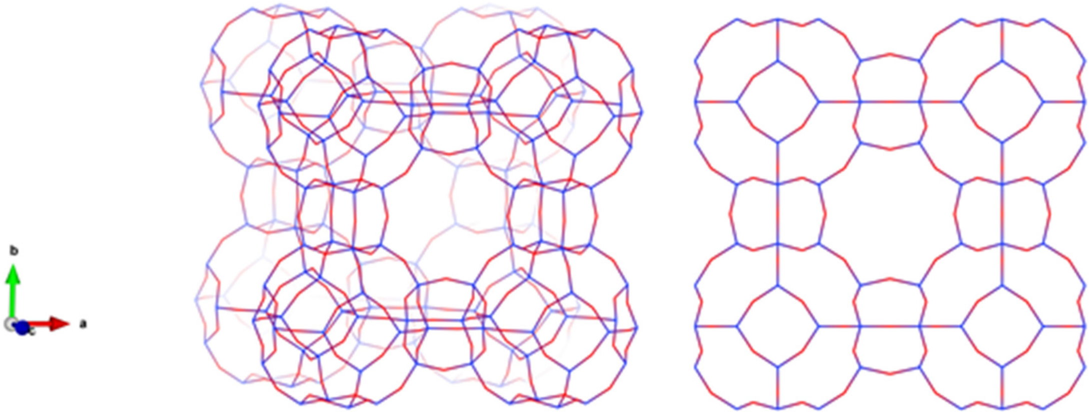

Figure S7.1. Structure of type A zeolites.

The cation presents in their structure dictated the pore size. For instance, if Na^+ is present, the pore opening (d_{pore}) is about 3.8 Å (4A zeolite), if it is K^+, the pore opening is about .3 Å (3A zeolite) and, if it is Ca^{2+}, the pore opening is 5 Å. Those pore opening define the size of molecules it may adsorb. When a molecule (in this case chitosan) is added to the zeolite'ssurface, it may cover some of the pores (Figure S7.2).

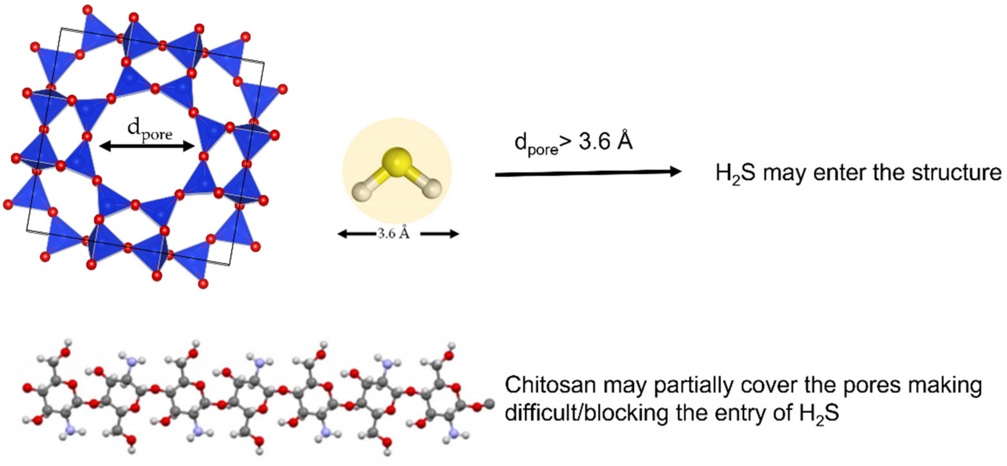

Figure S7.2. Schematic representation of the structure relationship between the zeolite, H_2S and chitosan.

References

1. Schäfer, K. The Virial Coefficients of Gases. A Critical Compilation. VonJ. H. Dymond undE. B. Smith (Oxford Science Research Papers No. 2). Clarendon Press: Oxford University Press, London 1969. 1. Aufl., XV, 231 S., geb. DM 21.60. *Angew. Chemie* **1970**, *82*, 642–642, doi:10.1002/ange.19700821521.
